# 2′,7′-Di­bromo­spiro­[cyclo­propane-1,9′-fluorene]

**DOI:** 10.1107/S1600536814014585

**Published:** 2014-06-25

**Authors:** Yue-yuan Xue, Yong-qi Qin

**Affiliations:** a Department of Chemistry and Chemical Engineering, Lvliang University, Lvliang, Shanxi 033001, People’s Republic of China; b Laboratory of Medicinal Chemistry, Lvliang University, Lvliang, Shanxi 033001, People’s Republic of China

**Keywords:** crystal structure

## Abstract

In the title compound, C_15_H_10_Br_2_, each mol­ecule is situated on special postion *mm*, so the asymmetric unit contains one-quater of a mol­ecule. The 2,7-di­bromo-9*H*-fluorene fragment and three spiro­cyclo­propane C atoms lie on different planes, which are perpendicular to each other. In the crystal, π–π inter­actions between aromatic rings [inter­centroid distance = 3.699 (3) Å] pack the mol­ecules into stacks extending in [001].

## Related literature   

For electroluminescence properties of fluorene derivatives, see: Cho *et al.* (2007 ▶); Jiang *et al.* (2005 ▶); Wei *et al.* (2008 ▶). For the crystal structures of related compounds, see: Jason *et al.* (1981 ▶); Wang *et al.* (2007 ▶).
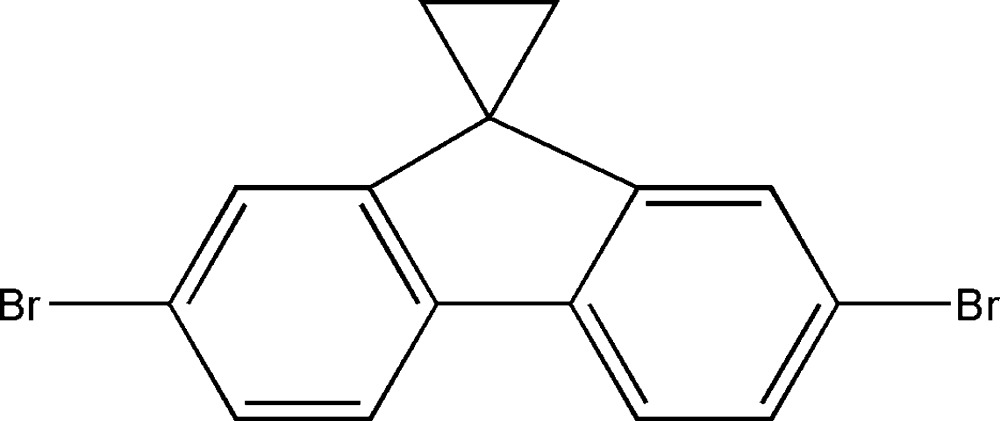


## Experimental   

### 

#### Crystal data  

C_15_H_10_Br_2_
*M* *_r_* = 350.05
Orthorhombic, 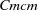

*a* = 16.9485 (17) Å
*b* = 11.0619 (11) Å
*c* = 6.8127 (10) Å
*V* = 1277.3 (3) Å^3^
*Z* = 4
Mo *K*α radiation
μ = 6.32 mm^−1^
*T* = 293 K
0.30 × 0.20 × 0.20 mm

#### Data collection  

Bruker SMART CCD area-detector diffractometer
3276 measured reflections
640 independent reflections
471 reflections with *I* > 2σ(*I*)
*R*_int_ = 0.155

#### Refinement  

*R*[*F* ^2^ > 2σ(*F* ^2^)] = 0.039
*wR*(*F* ^2^) = 0.100
*S* = 0.98
640 reflections
53 parameters
H-atom parameters constrained
Δρ_max_ = 0.30 e Å^−3^
Δρ_min_ = −0.68 e Å^−3^

### 

Data collection: *SMART* (Bruker, 2007 ▶); cell refinement: *SAINT* (Bruker, 2007 ▶); data reduction: *SAINT*; program(s) used to solve structure: *SHELXS97* (Sheldrick, 2008 ▶); program(s) used to refine structure: *SHELXL97* (Sheldrick, 2008 ▶); molecular graphics: *SHELXTL* (Sheldrick, 2008 ▶); software used to prepare material for publication: *SHELXTL*.

## Supplementary Material

Crystal structure: contains datablock(s) I. DOI: 10.1107/S1600536814014585/cv5463sup1.cif

Structure factors: contains datablock(s) I. DOI: 10.1107/S1600536814014585/cv5463Isup2.hkl

Supporting information file. DOI: 10.1107/S1600536814014585/cv5463Isup3.cml

CCDC reference: 1009321

Additional supporting information:  crystallographic information; 3D view; checkCIF report

## Figures and Tables

**Fig. 1.**
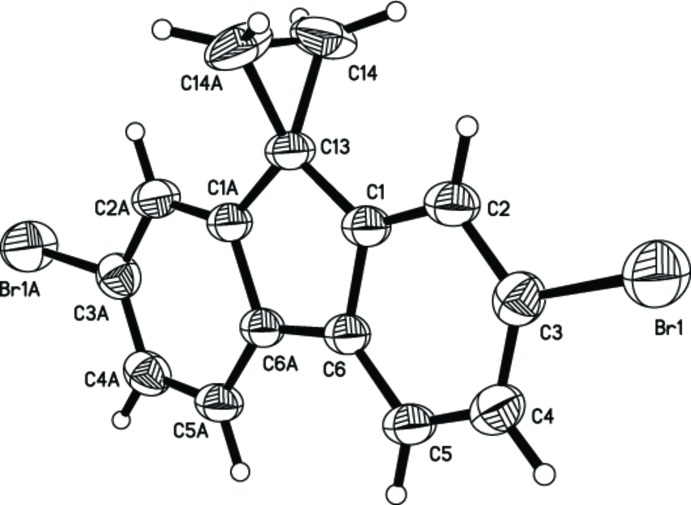
The molecular structure of (I) showing the atomic numbering and 40% probability displacement ellipsoids.

## supplementary crystallographic information

### S1. Comment

Fluorene derivatives have a wide range of applications in electroluminescence materials (Jiang *et al.*, 2005; Wei *et al.*, 2008) because of their good thermal, light and chemical stability (Cho *et al.*, 2007). Herewith we present the title compound (I), which is a new derivative of fluorene.

In (I) (Fig. 1), all bond lengths and angles are normal and comparable with those observed in the related spiro(cyclopropane-1,9'-(9H)fluorene) (Jason *et al.*, 1981) and 2',7'-diiodospiro(cyclopropane-1,9'-fluorene) (Wang, *et al.*, 2007). In (I), the 2,7-dibromo-9*H*-fluorene fragment and three carbon atoms of spirocyclopropane lie on different planes *m*, which are perpendicular to each other, so asymmetric unit contains one fourth of the molecule.

In the crystal, π–π interactions between the aromatic rings [intercentroid distance of 3.699 (3) Å] pack molecules into stacks extended in [001].

### S2. Experimental

The title compound was prepared by the reaction of 2,7-dibromo-9*H*-fluorene(0.01 mol), 1,2-dibromethane(0.01 mol) and KOH(0.03 mol) in 1,4-dioxane(20 ml) at 358 K for 3 h. Single crystals suitable for X-ray measurements were obtained by recrystallization from ethanol at room temperature.

### S3. Refinement

H atoms were fixed geometrically and allowed to ride on their parent atoms, with C—H distances = 0.93–0.97 Å; and with *U*_iso_(H) = 1.2*U*_eq_(C).

### Crystal data

**Table:**

C_15_H_10_Br_2_	*D*_x_ = 1.820 Mg m^−^^3^
*M**_r_* = 350.05	Mo *K*α radiation, λ = 0.71073 Å
Orthorhombic, *C**m**c**m*	Cell parameters from 933 reflections
*a* = 16.9485 (17) Å	θ = 2.2–25.0°
*b* = 11.0619 (11) Å	µ = 6.32 mm^−^^1^
*c* = 6.8127 (10) Å	*T* = 293 K
*V* = 1277.3 (3) Å^3^	Block, colourless
*Z* = 4	0.30 × 0.20 × 0.20 mm
*F*(000) = 680	

### Data collection

**Table:**

Bruker SMART CCD area-detector diffractometer	*R*_int_ = 0.155
Radiation source: fine-focus sealed tube	θ_max_ = 25.0°, θ_min_ = 2.2°
phi and ω scans	*h* = −20→17
3276 measured reflections	*k* = −13→12
640 independent reflections	*l* = −8→7
471 reflections with *I* > 2σ(*I*)	

### Refinement

**Table:**

Refinement on *F*^2^	0 restraints
Least-squares matrix: full	Hydrogen site location: difference Fourier map
*R*[*F*^2^ > 2σ(*F*^2^)] = 0.039	H-atom parameters constrained
*wR*(*F*^2^) = 0.100	*w* = 1/[σ^2^(*F*_o_^2^) + (0.0438*P*)^2^] where *P* = (*F*_o_^2^ + 2*F*_c_^2^)/3
*S* = 0.98	(Δ/σ)_max_ < 0.001
640 reflections	Δρ_max_ = 0.30 e Å^−^^3^
53 parameters	Δρ_min_ = −0.68 e Å^−^^3^

### Special details

**Table:**

Geometry. All e.s.d.'s (except the e.s.d. in the dihedral angle between two l.s. planes) are estimated using the full covariance matrix. The cell e.s.d.'s are taken into account individually in the estimation of e.s.d.'s in distances, angles and torsion angles; correlations between e.s.d.'s in cell parameters are only used when they are defined by crystal symmetry. An approximate (isotropic) treatment of cell e.s.d.'s is used for estimating e.s.d.'s involving l.s. planes.

### Fractional atomic coordinates and isotropic or equivalent isotropic displacement parameters (Å^2^)

**Table:**

	*x*	*y*	*z*	*U*_iso_*/*U*_eq_	
Br1	0.31110 (3)	0.12115 (5)	0.7500	0.0746 (4)	
C1	0.0691 (3)	0.1592 (4)	0.7500	0.0396 (11)	
C2	0.1489 (3)	0.1851 (4)	0.7500	0.0470 (12)	
H2	0.1666	0.2647	0.7500	0.056*	
C3	0.2011 (3)	0.0894 (5)	0.7500	0.0478 (12)	
C4	0.1763 (3)	−0.0277 (4)	0.7500	0.0506 (12)	
H4	0.2130	−0.0901	0.7500	0.061*	
C5	0.0965 (3)	−0.0541 (4)	0.7500	0.0450 (12)	
H5	0.0792	−0.1339	0.7500	0.054*	
C6	0.0429 (2)	0.0393 (4)	0.7500	0.0380 (10)	
C13	0.0000	0.2412 (5)	0.7500	0.0424 (16)	
C14	0.0000	0.3609 (3)	0.6418 (11)	0.0725 (18)	
H14	0.0485	0.3846	0.5754	0.087*	

### Atomic displacement parameters (Å^2^)

**Table:**

	*U*^11^	*U*^22^	*U*^33^	*U*^12^	*U*^13^	*U*^23^
Br1	0.0506 (4)	0.0657 (5)	0.1074 (6)	0.0008 (3)	0.000	0.000
C1	0.054 (3)	0.0309 (19)	0.034 (2)	−0.001 (2)	0.000	0.000
C2	0.056 (3)	0.034 (2)	0.051 (3)	−0.004 (2)	0.000	0.000
C3	0.047 (3)	0.049 (3)	0.047 (3)	0.005 (2)	0.000	0.000
C4	0.059 (3)	0.046 (3)	0.047 (3)	0.012 (2)	0.000	0.000
C5	0.063 (3)	0.031 (2)	0.041 (3)	0.003 (2)	0.000	0.000
C6	0.052 (2)	0.032 (2)	0.031 (2)	0.0006 (19)	0.000	0.000
C13	0.051 (4)	0.028 (3)	0.048 (4)	0.000	0.000	0.000
C14	0.054 (3)	0.039 (3)	0.125 (5)	0.000	0.000	0.030 (3)

### Geometric parameters (Å, º)

**Table:**

Br1—C3	1.897 (5)	C5—C6	1.375 (6)
C1—C2	1.383 (6)	C5—H5	0.9299
C1—C6	1.398 (6)	C6—C6^i^	1.454 (8)
C1—C13	1.481 (6)	C13—C1^i^	1.481 (6)
C2—C3	1.380 (7)	C13—C14	1.516 (7)
C2—H2	0.9300	C13—C14^ii^	1.516 (7)
C3—C4	1.361 (7)	C14—C14^ii^	1.475 (14)
C4—C5	1.384 (6)	C14—H14	0.9741
C4—H4	0.9299		
			
C2—C1—C6	120.5 (4)	C4—C5—H5	120.5
C2—C1—C13	130.3 (4)	C5—C6—C1	120.2 (4)
C6—C1—C13	109.3 (4)	C5—C6—C6^i^	131.3 (3)
C3—C2—C1	117.9 (4)	C1—C6—C6^i^	108.5 (3)
C3—C2—H2	121.3	C1^i^—C13—C1	104.5 (5)
C1—C2—H2	120.8	C1^i^—C13—C14	122.36 (19)
C4—C3—C2	122.1 (5)	C1—C13—C14	122.36 (19)
C4—C3—Br1	118.7 (4)	C1^i^—C13—C14^ii^	122.36 (19)
C2—C3—Br1	119.2 (4)	C1—C13—C14^ii^	122.36 (19)
C3—C4—C5	120.2 (4)	C14—C13—C14^ii^	58.2 (6)
C3—C4—H4	120.0	C14^ii^—C14—C13	60.9 (3)
C5—C4—H4	119.8	C14^ii^—C14—H14	117.6
C6—C5—C4	119.1 (4)	C13—C14—H14	117.4
C6—C5—H5	120.4		
			
C6—C1—C2—C3	0.0	C2—C1—C6—C6^i^	180.0
C13—C1—C2—C3	180.0	C13—C1—C6—C6^i^	0.0
C1—C2—C3—C4	0.0	C2—C1—C13—C1^i^	180.0
C1—C2—C3—Br1	180.0	C6—C1—C13—C1^i^	0.0
C2—C3—C4—C5	0.0	C2—C1—C13—C14	35.1 (4)
Br1—C3—C4—C5	180.0	C6—C1—C13—C14	−144.9 (4)
C3—C4—C5—C6	0.0	C2—C1—C13—C14^ii^	−35.1 (4)
C4—C5—C6—C1	0.0	C6—C1—C13—C14^ii^	144.9 (4)
C4—C5—C6—C6^i^	180.0	C1^i^—C13—C14—C14^ii^	110.6 (3)
C2—C1—C6—C5	0.0	C1—C13—C14—C14^ii^	−110.6 (3)
C13—C1—C6—C5	180.0		

Symmetry codes: (i) −*x*, *y*, *z*; (ii) *x*, *y*, −*z*+3/2.
